# Restricting Glutamine Uptake Enhances NSCLC Sensitivity to Third-Generation EGFR-TKI Almonertinib

**DOI:** 10.3389/fphar.2021.671328

**Published:** 2021-05-14

**Authors:** Yaming Liu, Xianming Ge, Jinlong Pang, Yuhan Zhang, Hao Zhang, Hongyan Wu, Fangtian Fan, Hao Liu

**Affiliations:** ^1^ Faculty of Pharmacy, Bengbu Medical College, Bengbu, China; ^2^ Department of Pharmacy, Bengbu Third People’s Hospital, Bengbu, China; ^3^ Institute of Biomedical Technology, Jiangsu Vocational College of Medicine, Yancheng, China

**Keywords:** almonertinib, SLC1A5, apoptosis, autophagy, EGFR-TKI

## Abstract

The emergence of secondary resistance is the main failure cause of epidermal growth factor receptor-tyrosine kinase inhibitors (EGFR-TKIs) as a targeted therapy for non-small cell lung cancer (NSCLC). EGFR mutations of NSCLC cells can markedly increase glutamine transporter (SLC1A5) expression, thereby increasing glutamine metabolism. Glutamine metabolites can activate EGFR downstream signals, including mTOR, ERK1/2, STAT3, etc., which is an important cause for the decreased sensitivity of NSCLC to EGFR-TKIs. CCK8 and Annexin V/PI assays were conducted to detect the effects of Almonertinib and/or V9302 on the proliferation and apoptosis of NSCLC cells. Proteomics was used to determine the effect of Almonertinib on energy metabolism-related proteins in NSCLC. siRNA transfection was performed to study the effect of SLC1A5 down-regulation on cell proliferation. In addition, the effects of drugs on colony formation capacity were determined by colony formation assay. Immunofluorescence and Western blot were utilized to detect the apoptosis- and autophagy-related proteins expression. DAPI staining was utilized to detect the effect of drugs on the nucleus. Transmission electron microscope was used to observe the changes of submicroscopic structure such as autophagosomes and nucleus of cells. mCherry-GFP-LC3B tandem fluorescent protein was to used to detect the level of autophagy flux. Tumor-bearing nude mouse model was utilized to detect the effect of V9302 on the anti-tumor effect of Almonertinib *in vivo*. As a result, Almonertinib suppressed H1975 and A549 cell proliferation depended on its dosage and treatment duration, and it also induced apoptosis. A549 cells with wild-type EGFR had lower sensitivity to Almonertinib. The expression of SLC1A5 was up-regulated by stimulating with low concentration of Almonertinib in NSCLC cells. SLC1A5 was highly expressed in A549 cells with wild-type EGFR. Glutamine deletion or SLC1A5 inhibition/silencing inhibited the proliferation of NSCLC cells, and decreased cellular glutamine uptake. The combination of SLC1A5 inhibitor V9302 and Almonertinib had a synergistic inhibitory effect on the proliferation of NSCLC. V9302 enhanced the effect of Almonertinib in apoptosis-inducing in NSCLC cells. The combination of V9302 and Almonertinib might induce apoptosis by inhibiting autophagy.

## Introduction

Lung cancer (LC) currently ranks first in terms of incidence and mortality of all types of malignancies worldwide, which severely affects human health. According to the global cancer statistics in 2018, the incidence of lung cancer accounted for 11.6% overall cases, and the mortality accounted for 18.4% of all cancer-associated mortality (Bray et al., 2018). Epidemiology shows that approximately 30–40% of non-small cell lung cancer (NSCLC) patients have epidermal growth factor receptor (EGFR) mutations (Dizon et al., 2016). As confirmed in clinical studies, patients carrying sensitive EGFR mutations benefit from the small-molecule selective tyrosine kinase inhibitor (TKI), with longer progression-free survival (PFS) from 4–6 months of standard platinum-based double-drug chemotherapy to 9–13 months (Minari et al., 2016). However, most NSCLC patients still inevitably have attenuated sensitivity after receiving EGFR-TKIs for 8–16 months (Makinoshima et al., 2014), which has become a main issue in treating NSCLC. Therefore, seeking therapeutic strategies to increase the sensitivity to EGFR-TKIs of tumor cells is urgently needed.

Glutamine represents the amino acid with the highest abundance in blood, which is also a cellular nitrogen source and carbon or nitrogen donor (Deberardinis and Cheng, 2010; Villar et al., 2015). Glutamine is widely used for various biochemical functions, including cellular redox homeostasis, protein synthesis, purine synthesis, as well as citric acid or Krebs cycle (Marzi et al., 2016). Research had shown metabolic profiling analysis of lung cancer tissues, revealing that Glutathione (GSH) level was higher in lung cancer tissue than healthy lung tissue (Gamcsik et al., 2012). Massive accumulation of GSH leads to the super-antioxidant capacity of tumor cells, which exerts a vital part in radiochemotherapy sensitivity (Hanigan, 2014). The increased metabolism of glutamine in tumor cells leads to the accumulation of corresponding metabolites, and the metabolites can activate the EGFR downstream signals, including mechanistic Target of Rapamycin (mTOR) (Yang et al., 2017), ERK1/2, STAT3, etc. (Villar et al., 2015; Yang et al., 2017; Vanhove et al., 2019), which become an important reason of the decreased NSCLC sensitivity to EGFR-TKIs. Therefore, we speculate that restricting glutamine intake may increase the NSCLC sensitivity to EGFR-TKIs and delay drug resistance.

**Figure:**
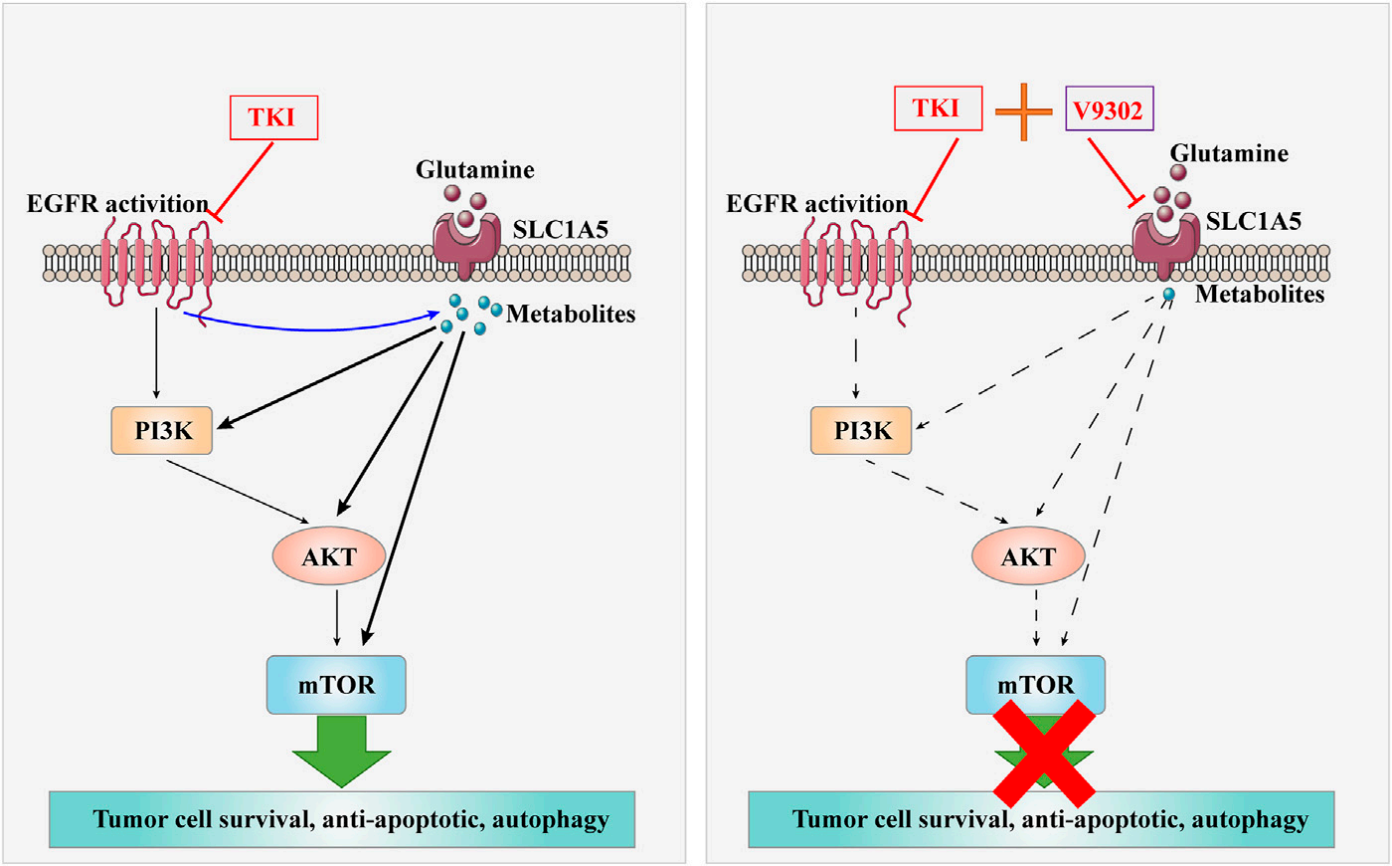
**       Graphical Abstract** **|** The combination of V9302 and Almonertinib might induce apoptosis of non-small cell lung cancer cells.

The influx of glutamine needs to be mediated by the glutamine transporter on the cell membrane. Alanine-serine-cysteine transporter-2 (ASCT2 or SLC1A5) is a solute carrier 1 and a sodium-dependent transporter, which is responsible for over 50% of the transmembrane influx of glutamine by exchanging neutral amino acids through the cell membranes of peripheral tissues (Wang et al., 2015). Hassanein et al. confirmed that this receptor is overexpressed in squamous cell carcinoma, adenocarcinoma and neuroendocrine lung tumors (Hassanein et al., 2013; van Geldermalsen et al., 2016), which exerts a vital part in enhancingtumor development. SLC1A5 is considered to be the main entrance of glutamine. Inhibition of SLC1A5 effect can lead to the reduction of tumor cell growth and proliferation. Pharmacological SLC1A5 inhibitor l-glutamyl-p-nitroanilide (GPNA) and siRNA silencing can induce cell cycle arrest at G1 stage and decrease cell activity by blocking mTOR signal transduction in lung cancer cells (Hassanein et al., 2013).

Almonertinib is the third-generation EGFR small molecule inhibitor for NSCLC recently approved by the China Food and Drug Administration. Almonertinib can inhibit the phosphorylation of mutant EGFR to block the downstream signal transduction by irreversibly binding to mutant EGFR, thereby inhibiting proliferation and inducing apoptosis of NSCLC cells. However, it is not clear whether interference with abnormal glutamine metabolism of tumors can affect the sensitivity of Almonertinib. As a result, this work aimed to explore whether glutamine intake restriction could affect the sensitivity of NSCLC cells to Almonertinib, which can not only provide the theoretical basis to delay post-marketing drug resistance, but provide a sensitization strategy for possible decreased sensitivity of long-term clinical application of third-generation EGFR small molecule inhibitors.

## Materials and Methods

### Cells and Cell Culture

NSCLC cell lines (H1975 and A549) and Normal lung epithelial cells (BEAS-2B) were provided by Shanghai Cell Bank and cultivated within the Roswell Park Memorial Institute Medium (RPMI) 1,640 containing 10% fetal bovine serum (FBS), as well as 100 µg/ml penicillin-streptomycin. T790m mutant cell line H1975 was constructed at our laboratory and utilized in this experimental study *in vitro* and *in vivo*.

### Drug Treatment and Cell Viability Assay

The H1975,A549 and BEAS-2B cell lines were inoculated into the 96-well plates at 1 × 10^4^/well and into the 100-mm dishes at 1 × 10^6^/dish. After incubation for 24 h, Almonertinib and/or SLC1A5 inhibitor V9302 (MedChemexpress, NJ, United States) at diverse doses (Jiangsu Haoseh Pharmaceutical Group Co., Ltd.) was used to co-incubate H1975 and A549 cells for 24 h. After incubation, CCK-8 assay (Dojin Chemical, Kumamoto, Japan) was conducted to determine cell viability in accordance with specific instructions.

### Proteomics

After drug treatment for 24 h, cells were carefully scraped uisng a cell scraper. Cell lysate was transferred into a centrifuge tube, followed by freezing within the liquid nitrogen as well as preservation under −80°C. Proteomics was performed by Shanghai Biotree Biomedical Technology Co., Ltd.

### Colony Formation Assay

To carry out colony formation assay, we cultivated the tumor cells within Dulbecco’s Modified Eagle Medium (DMEM) containing 10% Fasting Blood Sugar (FBS) and SphereMax (Wako) within the 96-well ultra-low attachment plate (Corning, NY, United States) at 100 cells/well for a period of 10 days. Thereafter, the colony number (size >100 µM) was determined under the microscope.

### Cell Death by Flowmetry (Annexin V-FITC/PI)

After drug treatment for 24 h, cells were carefully scraped using a cell scraper. Cell lysate was added to the a centrifuge tube for cytometry using Annexin V-FITC/PI detection kit (Jiangsu Kaiji Biotechnology Co., Ltd.) in accordance with specific protocols.

### Cellular Autophagosome Formation by Transmission Electron Microscopy

After drug treatment for 24 h, the supernatant was discarded, and Phosphate Buffered Saline (PBS) was used to wash cells, followed by digestion. Cells were collected, followed by 6 min centrifugation at 12,000 rpm/min under 4°C and discarding of the upper layer of liquid. Cells were re-suspended with pre-cooled PBS, centrifuged, followed by discarding the supernatant. Cells were collected into a 1.5 ml centrifuge tube. The total cell volume should be approximately half of a soybean size. Afterward, 1 ml of 25% glutaraldehyde was added along the centrifuge tube wall for fixation and stored at 4°C. Samples were sent to Pathology Department of Anhui Provincial Hospital for examination, followed by image acquisition.

### Detection of Autophagic Flux by mCherry-GFP-LC3B Transfection

GFP-mCherry-LC3 tandem fluorescent protein is a fusion protein to specifically observe the level of autophagic flux. Yellow fluorescence in the cell (co-localization of green and red fluorescence) in the synthetic image indicated that the fusion protein was not fused with the lysosome or the pH value in the autolysosome was higher, further representing autophagic flux blocking. Red fluorescence in the cell in the synthetic image indicated the localization of fusion protein in the lysosome or autolysosome, suggesting the activation of autophagic flux. For specific protocol, in brief, mCherry-GFP-LC3B transfection plasmid was prepared by Shanghai Genepharma Company. Lipofectamine 2000 was used to transfect cells. After transfection, monoclone was selected for culture, intervened with drug for 24 h and mounted, followed by image acquisition by the laser confocal microscopy.

### Effects of Drug on Cellular Nuclei by DAPI Staining

After 24 h of drug treatment, PBS was used to rinse cells thrice, and 1 ml of the 4% paraformaldehyde (PFA) was used to fix cells for 30 min under ambient temperature. After removing the fixative, 1 ml PBS was used to rinse cells thrice, followed by addition of 1 ml of DAPI staining solution. After 5 min staining in dark, cells were rinsed thoroughly by PBS for four times and wrapped with silver paper, followed by image acquisition of live cell station.

### Immunofluorescence

The supernatant was discarded, cells were rinsed by pre-cooled PBS, followed by fixation using 1 ml 4% PFA for 30 min under ambient temperature. After removing the fixative, cells were subjected to PBS washing thrice and 5 min of permeabilization using Triton X-100 (0.2%). Cells were rinsed with PBS, blocked using 5% BSA solution for 30 min and incubated using the prepared primary antibody (dilution 1:100, 20 µl/well) overnight at 4°C. The next day, after removing primary antibody liquid, cells were rinsed by TPBS thrice, followed by incubation using 50 µl fluorescent secondary antibody at dark for 2 h. Then, cells were rinsed by TPBS thrice, stained by DAPI staining solution for 5 min, and rinsed by PBS four times (5 min each time), followed by image acquisition by live cell station.

### siRNA Transfection

The sequence of SLC1A5 siRNA was shown below:

**Table:** 

Negative control sense: 5′-UUC UCC GAA CGU GUC ACG UTT-3′
Antisense: 5′-ACG UGA CAC GUU CGG AGA ATT-3′
SLC1A5-homo-1,313 sense: 5′-CCU GGG CUU GGU AGU GUU UTT-3′
Antisense: 5′-AAA CAC UAC CAA GCC CAG GTT-3′
SLC1A5-homo-1,522 sense: 5′-GCC UUG GCA AGU ACA UUC UTT-3′
Antisense: 5′-AGA AUG UAC UUG CCA AGG CTT-3′
SLC1A5-homo-1,968 sense: 5′-GUC GAC CAU AUC UCC UUG ATT-3′
Antisense: 5′-UCA AGG AGA UAU GGU CGA CTT-3’.

Lipofectamine 2000 was used for cell transfection. And cells were subjected to relevant experimental protocol after 48 h.

### Western Blotting

After relevant treatments, we collected cells and rinsed them using cold PBS thrice. Thereafter, Radio Immuno Precipitation Assay (RIPA) lysis buffer (Beyotime, China) that contained 1% phenylmethylsulfonylfluoride (PMSF, Beyotime, China) was used to lyse cells. Supernatants were collected through 15 min of centrifugation of the whole-cell lysates at 12,000 rpm/min under 4°C. Weighed the tumor tissue 100–200 mg, ground it with liquid nitrogen in a mortar, added protein extract 500–1000 μl, inhaled it into a 1.5 ml sterilized EP tube, kept the EP tube containing protein extract in an ice bath for 20 min at 4°C, centrifuged at 14,000 rpm for 20 min, extracted the supernatant, and separately stored it in −80°C.Protein content was detected through Bicin Choninic Acid (BCA) assay. 12% Sodium Dodecyl Sulphate-Polyacrylamide Gel Electrophoresis (SDS-PAGE) was conducted to separate equivalent volumes of protein (50 mg), and the separated proteins were then transferred to the Polyvinylidene Fluoride (PVDF) membranes. Later, the membranes were blocked by 5% (w/v) skimmed milk powder within the Tris-buffered saline that contained 0.1% (v/v) Tween-20 (TBST) for 2 h for blocking the nonspecific binding sites, followed by overnight primary antibody incubation under 4°C. Membranes were washed by TBST, followed by 2 h of incubation using secondary antibodies under ambient temperature. After incubation, images were visualized by Bio-rad gel imaging system.

### Uptake of Glutamine

2.0 mM glutamine premix was prepared through blending 5 μl of 100 mM standard solution with 245 μl of distilled water. Thereafter, the standard solution was diluted according to the instructions. 20 μl of the standard solution was added into the transparent flat-bottom 96-well plate to prepare a standard curve. After drug treatment for 24 h, for preparation of every standard sample as well as sample well, we prepared the working reagents through blending 1 μl enzyme A, 1 μl enzyme B, 65 μl measurement buffer, 14 μl MTT and 2.5 μl NAD. For blank sample, the blank working reagent could be prepared through blending 1 μl enzyme B, 65 μl measurement buffer, 14 μl MTT and 2.5 μl NAD (without enzyme A). Eighty microliter working reagent was added into each standard sample and sample well. Eighty microliter blank working reagent was added to each blank sample well when appropriate, followed by board tapping for thorough mixing. After 40 min incubation under ambient temperature, we added 100 μL of stop reagent into every well. Cell samples were subjected to OD value at 565 nm, followed by calculation of glutamine content according to the standard curve.

### Xenograft Models of Nude Mice

All animal experiments were approved by the Ethics Committee for Animal Experiments of Bengbu Medical College (Permission Number: 2020-038) and were performed in accordance with the Guidelines for Laboratory Animal Experiments. The 6-8-week-old male BALB/c nude mice were provided by Shanghai Slac Biological Company. 5 × 10^5^ cells were suspended within the 100 µl DMEM, and the suspension was injected into the back of each mouse on day 0 in the subcutaneous approach. Vehicle or different doses of drugs were administered by intraperitoneal injection every other day. Each mouse was sacrificed on day 14. After subcutaneous tumor was formed, the tumor volume was measured and the vital signs of mice were carefully monitored. After 14 days of drug administration, blood samples were collected from mouse eyes and mice were sacrificed, followed by tumor excision. The liver and kidney of mice were fixed in 4% PFA and photographed. The blood samples were sent to the laboratory of the First Affiliated Hospital for liver and kidney toxicity testing. Each animal experiment gained approval from the Ethics Committee for Animal Experiments of Bengbu Medical College.

### Statistical Analysis

The values were means ± SD from two or three independent experiments. The significance of difference was determined by non-repeated measures Analysis of Variance (ANOVA) and Mann–Whitney *U*-test. A difference of *p* < 0.05 was deemed statistically significant.

## Results

### EGFR Mutant NSCLC Cells Were More Sensitive to Almonertinib Than EGFR Wild-Type Cells

**FIGURE 1:**
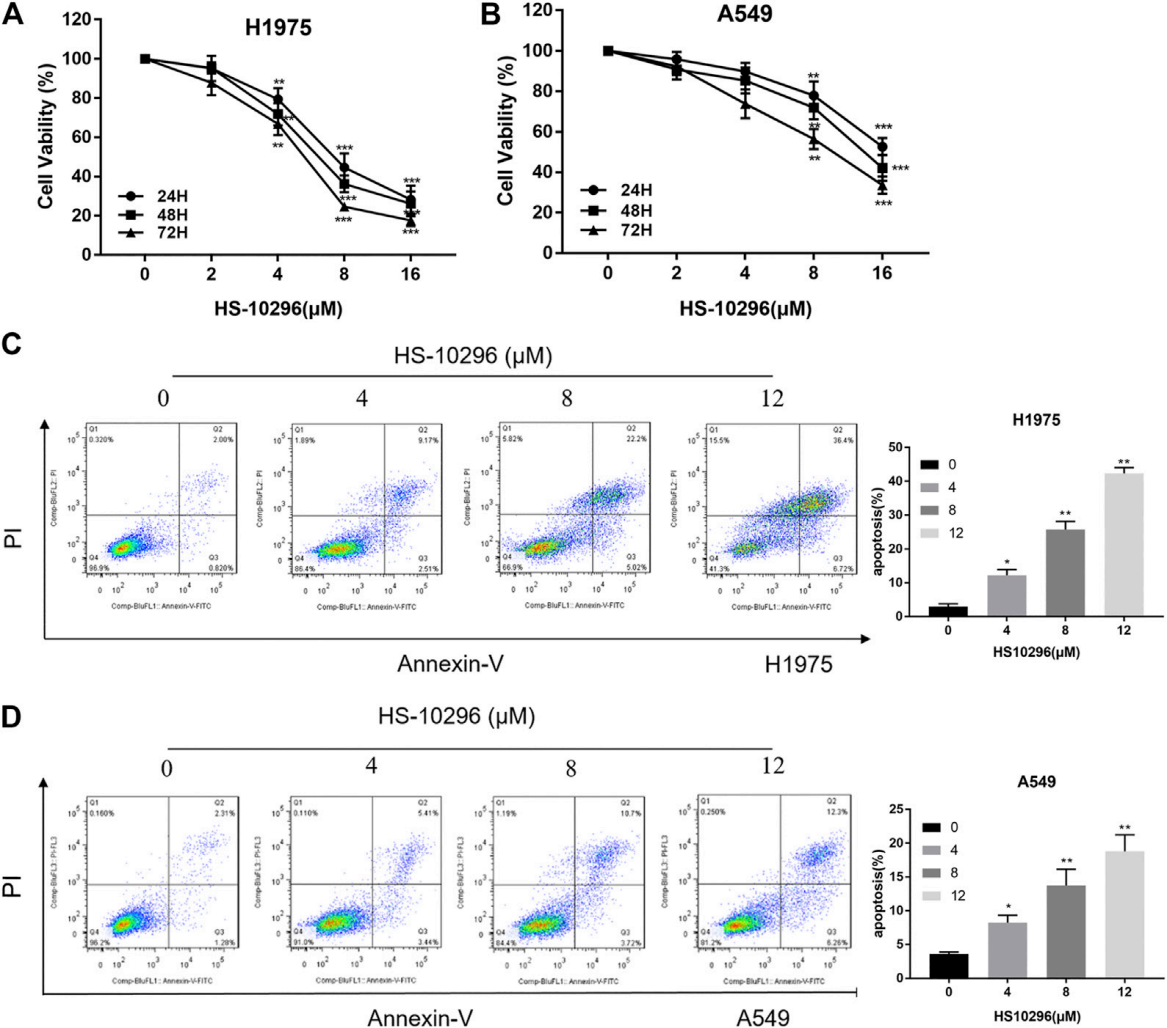
Effects of Almonertinib on the proliferation and apoptosis of NSCLC cells. **(A,B)** CCK8 assay was used to detect different concentrations of Almonertinib (2, 4, 8, and 16 µM) on H1975 and A549 cells for 24, 48, and 72 h. **(C,D)** Flow cytometry was used to detect the apoptosis rate of different concentrations of Almonertinib on H1975 and A549 cells after 24 h. Significance: *p < 0.05, **p < 0.01,***p < 0.001.

To test the inhibitory effect of Almonertinib on the proliferation of NSCLC cells, we selected EGFR wild-type (WT) cell line A549 and T790m mutant cell line H1975 to observe the effects of different concentrations of Almonertinib (2, 4, 8, and16 µM) on cell viability of two cell lines after 24, 48, and 72 h. CCK8 assay showed that the viability rate of EGFR mutant cell line H1975 was significantly decreased in a concentration-dependent manner. However, the viability rate of EGFR WT cell line A549 cells was not significantly decreased compared with H1975 (Figures 1A,B), indicating that EGFR WT NSCLC cells were not sensitive to Almonertinib. Furthermore, Annexin-V/PI flow cytometry was used to explore the effect of Almonertinib on the apoptosis of H1975 and A549 cells. Almonertinib increased the apoptosis rate of H1975 cells in a concentration-dependent manner. When the concentration of Almonertinib was 12 μM, the apoptotic rate of H1975 cell was 43.12%, however, the apoptotic rate of A549 cells was only 18.56% (Figures 1C,D).

### Almonertinib Increased the Expression of Glutamine Transporter in NSCLC Cells

**FIGURE 2:**
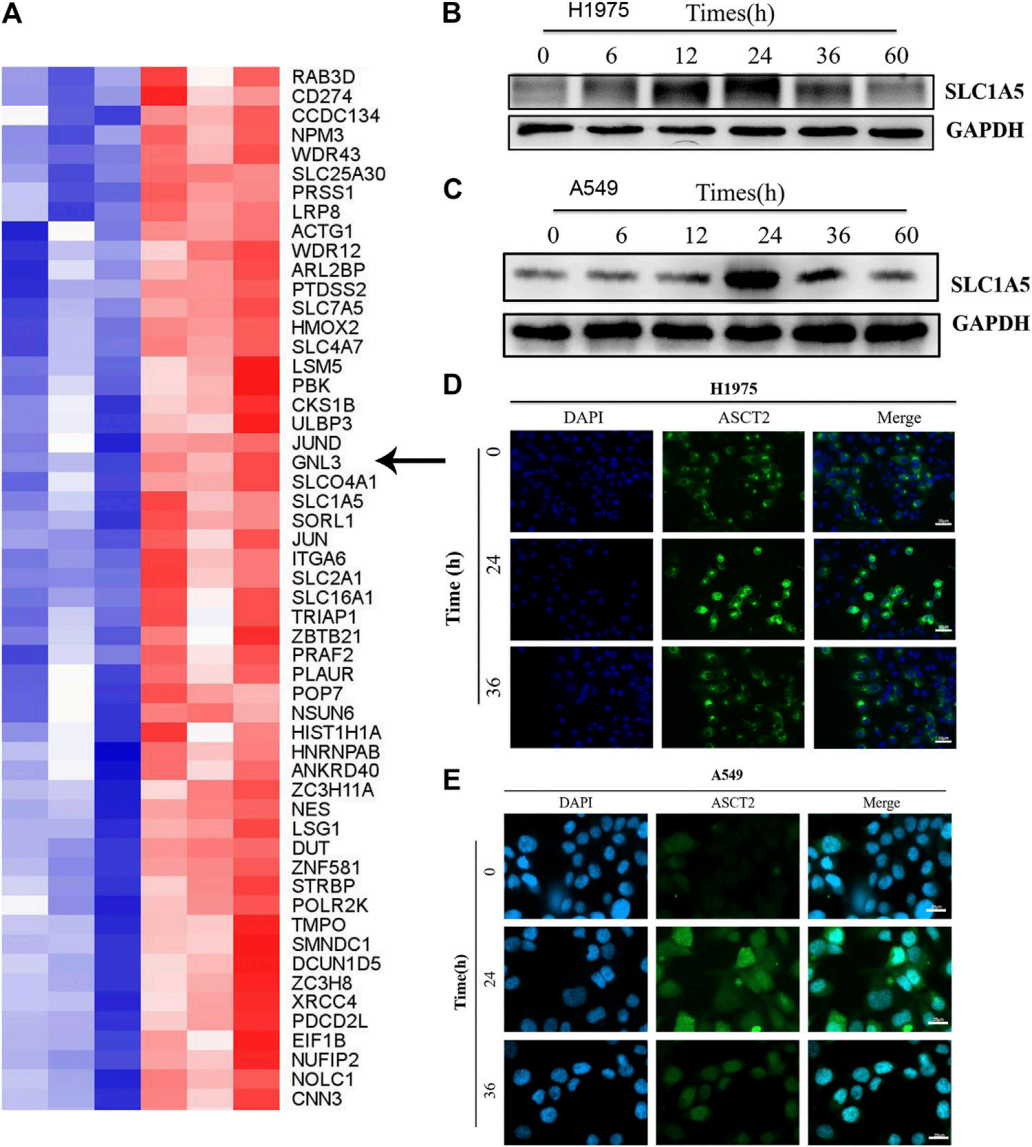
ASCT2 (SLC1A5) protein expression was up-regulated in Almonertinib-induced energy metabolic transport channels. **(A)** After stimulating H1975 cells with Almonertinib at IC50/2 (4 µM) for 18 h, the cells were collected and LC-MS was used to analysis differences in the expression of related proteins. The arrow shows the glutamine transporter SLC1A5. **(B,C)** Western blotting (WB) was used to examine the expression of SLC1A5 protein in H1975 cells stimulated by Almonertinib at different times. **(D,E)** Immunofluorescence was used to detect the expression of SLC1A5 protein in H1975 cells stimulated by Almonertinib at different times.

The effect of Almonertinib on energy metabolism-related transport channels was further evaluated in H1975 cells. LC-MS measurement revealed that the expression of ASCT2 (SLC1A5) in the Almonertinib group was significantly increased compared to the blank control group (Figure 2A), highlighting that SLC1A5 might affect the response of H1975 cells to Almonertinib. Western blotting (WB) showed that the protein expression of ASCT2 (SLC1A5) was gradually increased with the increasing administration time of Almonertinib on H1975 and A549 cells, reaching a peak at 24 h after drug administration (Figures 2B,C). To further validate the protein expression of ASCT2 (SLC1A5) after Almonertinib administration for different time, IF was performed to detect the expression of ASCT2 (SLC1A5) after stimulation of 4 µM Almonertinib on H1975 and A549 cells for 24 and 36 h. Consistently, IF also proved that the protein expression of ASCT2 (SLC1A5) was increased after Almonertinib treatment for 24 h (Figures 2D,E). The results of WB and IF were consistent with the results of proteomics.

### Effects of SLC1A5 Silencing or Suppression on Cell Proliferation and Glutamine Uptake in NSCLC

**FIGURE 3:**
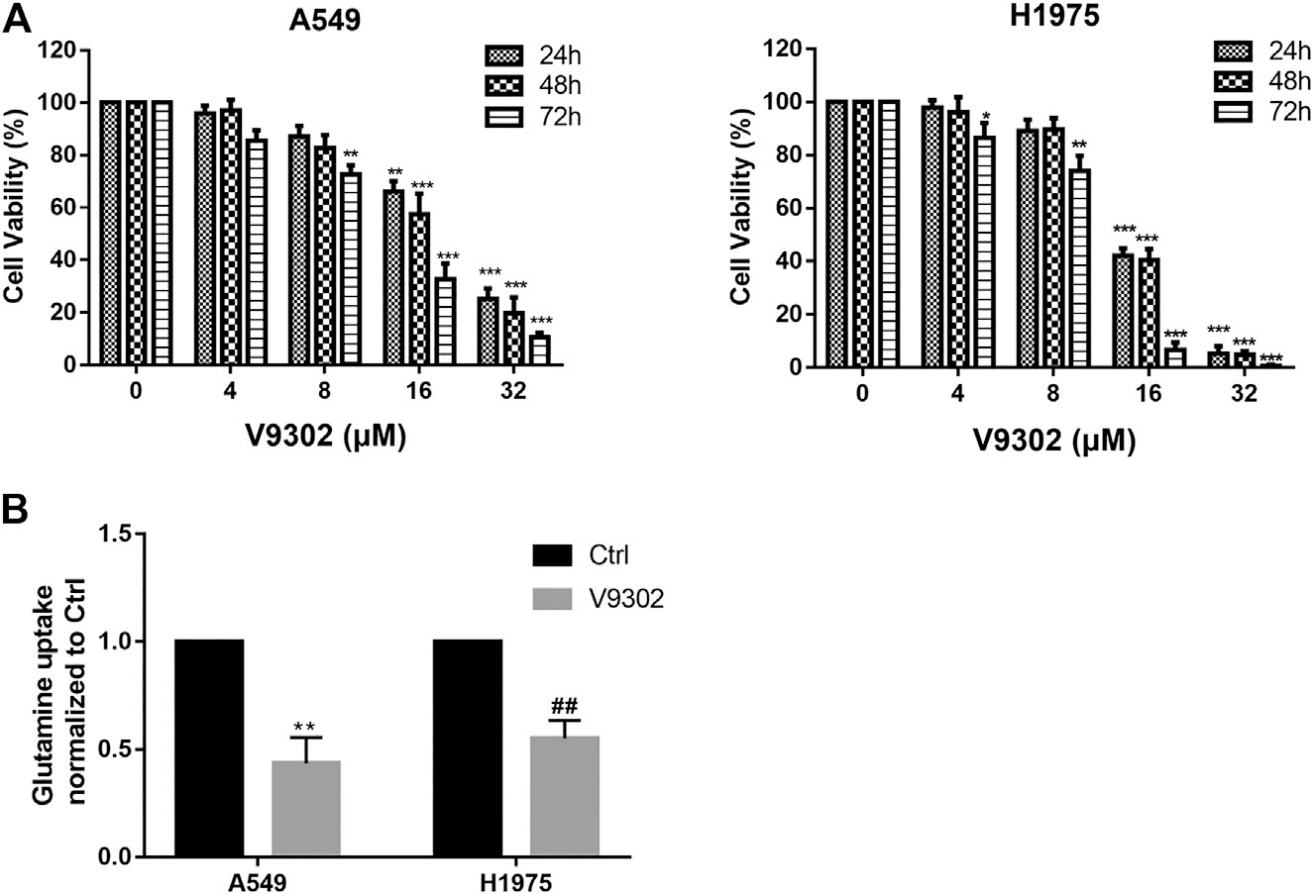
Effects of SLC1A5 inhibition or knockdown on cell proliferation and glutamine uptake in NSCLC cells. **(A)** CCK8 assay was used to detect different concentrations of V9302 on H1975 and A549 cells for 24, 48 and 72 h. **(B)** Inhibition SLC1A5 or knockdown of SLC1A5 can reduce the uptake of glutamine in NSCLC cells. Significance: *p < 0.05, **p < 0.01,***p < 0.001, ^##^ *p* < 0.01.

Relevant literature has reported that SLC1A5 was the most important transporter to absorb exogenous glutamine in various cells (including breast cancer, NSCLC, prostate cancer, etc.), and regulated tumor growth by controlling the cellular entry of glutamine (Hassanein et al., 2013; van Geldermalsen et al., 2016). V9302 is a competitive antagonist of transmembrane glutamine flux and can target the glutamine transporter SLC1A5 effectively and selectively. Therefore, different concentrations of V9302 (0∼32 µM) were used to treat two cell lines for 24, 48, and 72 h, followed by detection of cell viability by CCK8 assay. As shown in Figure 3A, V9302exerted a potent inhibitory effect on proliferation of both cell lines in time-dependent and dose-dependent manners. To verify that SLC1A5 inhibition could effectively inhibit cellular uptake of glutamine, the cellular glutamine uptake assay was performed. As shown in Figure 3B, V9302 (10 mM) decreased the absorption of glutamine in A549 and H1975 cells compared with the control group. This result indicated that V9302 decreased the uptake of glutamine by inhibiting the activity of SLC1A5 in NSCLC cells.

### Effect of V9302 on Almonertinib-Induced Proliferative Inhibition in NSCLC

**FIGURE 4:**
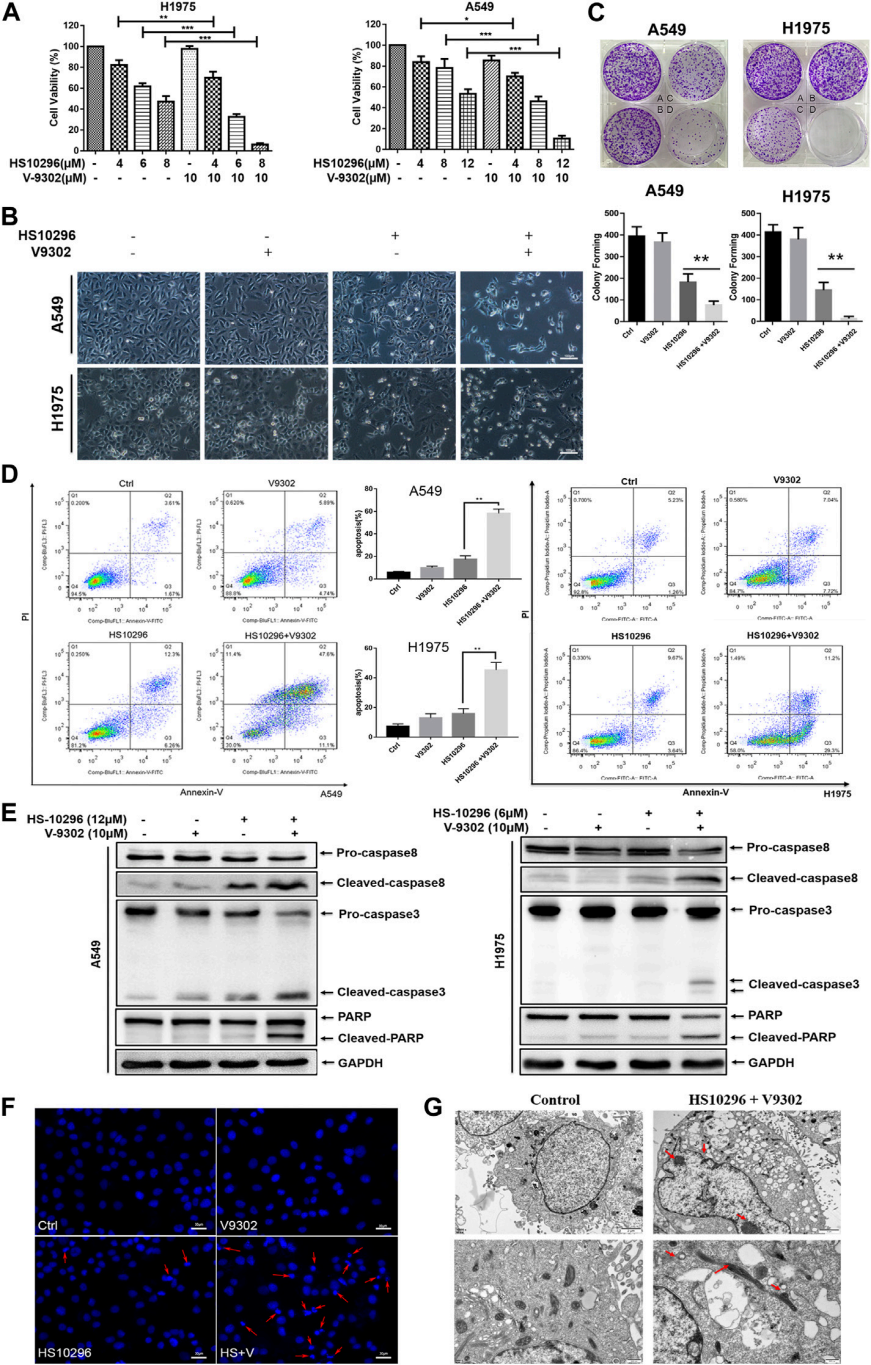
V9302 enhanced the inhibitory effect of Almonertinib on the proliferation of NSCLC cells. **(A)** The effect of different concentrations of Almonertinib and 10 µM V9302 on cells alone or in combination was used for 24 h to test the effect of H1975 or A549 cell viability by CCK8. **(B)** Cell morphological changes observed under an inverted microscope after 12 μM (or 6 μM) Almonertinib and 10 μM V9302 were used separately or in combination for 24 h. **(C)** Effects of Almonertinib and V9302 alone or in combination on the colony forming ability. A549 cells (A: blank group B: 1 µM V9302 C: 1.2 µM Almonertinib D: 1 µM V9302 and 1.2 µM Almonertinib); H1975 cells (A: blank group B: 1 µM V9302 C: 0.8 µM Almonertinib D: 1 µM V9302 and 0.8 µM Almonertinib). **(D)** After the intervention of Almonertinib and V9302 alone or in combination for 24 h, the apoptosis rate of A549 cells was detected by double staining. **(E)** Protein expression of caspase8, caspase3 and PARP. The effects of Almonertinib and V9302 alone or in combination on the expression of A549 or H1975 cells for 24 h were detected by WB. **(F)** DAPI fluorescence staining was used to observe the nuclear changes of H1975 cells after Almonertinib and V9302 were treated alone or in combination for 24 h. **(G)** The submicroscopic structure of H1975 cells was observed by electron microscopy after treat with 6 µM Almonertinib combined with 10 µM V9302 for 12 h. Significance: *p < 0.05, **p < 0.01.

To further illustrate the significance of SLC1A5 on the sensitivity of NSCLC to Almonertinib, the effect of SLC1A5 inhibition on the response of NSCLC to Almonertinib was further studied. According to the CCK8 assay results of V9302, we first selected three different concentrations of V9302 (8, 10, and 12 µM) in combination with different concentrations of Almonertinib for preliminary experiments. Accordingly, we finally chose 10 µM of V9302 combined with different concentrations of Almonertinib. CCK8 assay and microscopic cell morphology showed that V9302 exerted a significant synergistic effect on Almonertinib (CompuSyn software was used to calculate that the CI was less than one of the three concentrations of Almonertinib combined with 10 µM V9302) (Figure 4A). We further observed the cell morphology alterations of Almonertinib and V9302 after single or combined administration. As a result, a large number of vacuoles appeared in A549 cells in the Almonertinib single-treatment group under the microscope; the appearance of H1975 cells appeared was swollen, without clear outline, and part of cellular morphology turned into a spindle shape. While the number of cells was significantly reduced in the two-drug combination group, with significantly increased cellular gap, round and bright of cells and floating in the culture medium (Figure 4B). In addition, colony formation assay is one of the indicators to evaluate the inhibitory effect of drugs on tumor cell proliferation. Colony formation assay showed that the combined use of Almonertinib and V9302 greatly decreased the number of formed colonies (Figure 4C). Altogether, the above findings suggested that the addition of SLC1A5 inhibitor V9302 could enhance the sensitivity of cells to Almonertinib. It is interesting that Almonertinib and V9302 used alone or in combination will not affect the proliferation of normal epithelial cells BEAS-2B at equal concentrations (Supplementary Figure S1).

We further investigated whether the synergistic inhibition of Almonertinib combined with V9302 on cells was associated with apoptosis. To this end, flow cytometry was used to detect the apoptosis rate of cells in different administration groups by Annexin-V/PI assay (Figure 4D). Almonertinib (12 µM) and V9302 (10 µM) were used alone or in combination to interfere with A549 cells. Similarly, Almonertinib (6 µM) and V9302 (10 µM) were used alone or in combination to interfere with H1975 cells. Cells were subjected to flow cytometry after 24 h. As a result, the apoptosis rate of the combined drug group was significantly increased compared with the single drug group of Almonertinib, indicating that V9302 enhanced the apoptosis-inducing effect of Almonertinib. We subsequently analyzed several apoptosis markers by Western blot, including the cleavage of caspase8 and caspase3 and the enzymolysis of the substrate Poly-ADP Ribose Polymerase (PARP). As shown in Figure 4E, Almonertinib combined with V9302 treatment increased the cleavage of caspase8 and PARP in A549 and H1975 cells; similarly, the cleavage of caspase 8 was also increased. Additionally, we also observed the changes in the nucleus of different drug groups (the same dose as above) 24 h by live cell station. DAPI IF staining showed that the nucleus in the blank control group was intact and the chromatin was evenly distributed; a small amount of cellular nuclei fragmentation and chromatin shrinkage could be observed in Almonertinib single-use group; while massive nuclei fragmentation and chromatin shrinkage appeared in the Almonertinib and V9302 combination group, and fluorescence of the nucleus changed from uniform weak light to blue strong light under microscope, indicating the occurrence of apoptosis (Figure 4F). To further validate that the combination of Almonertinib and V9302 induced cell apoptosis, TEM was used to observe the submicroscopic structure of cells. As shown in Figure 4G, the arrow indicated chromatin aggregation and edge aggregation in the nucleus and the shrinkage of the nuclear membrane, swollen mitochondria, suggesting early apoptosis. The above assays suggested that V9302 could enhance the effect of Almonertinib in inducing apoptosis of NSCLC cells.

### Effect of V9302 on the Anti-Tumor Effect of Almonertinib in Nude Mice

**FIGURE 5:**
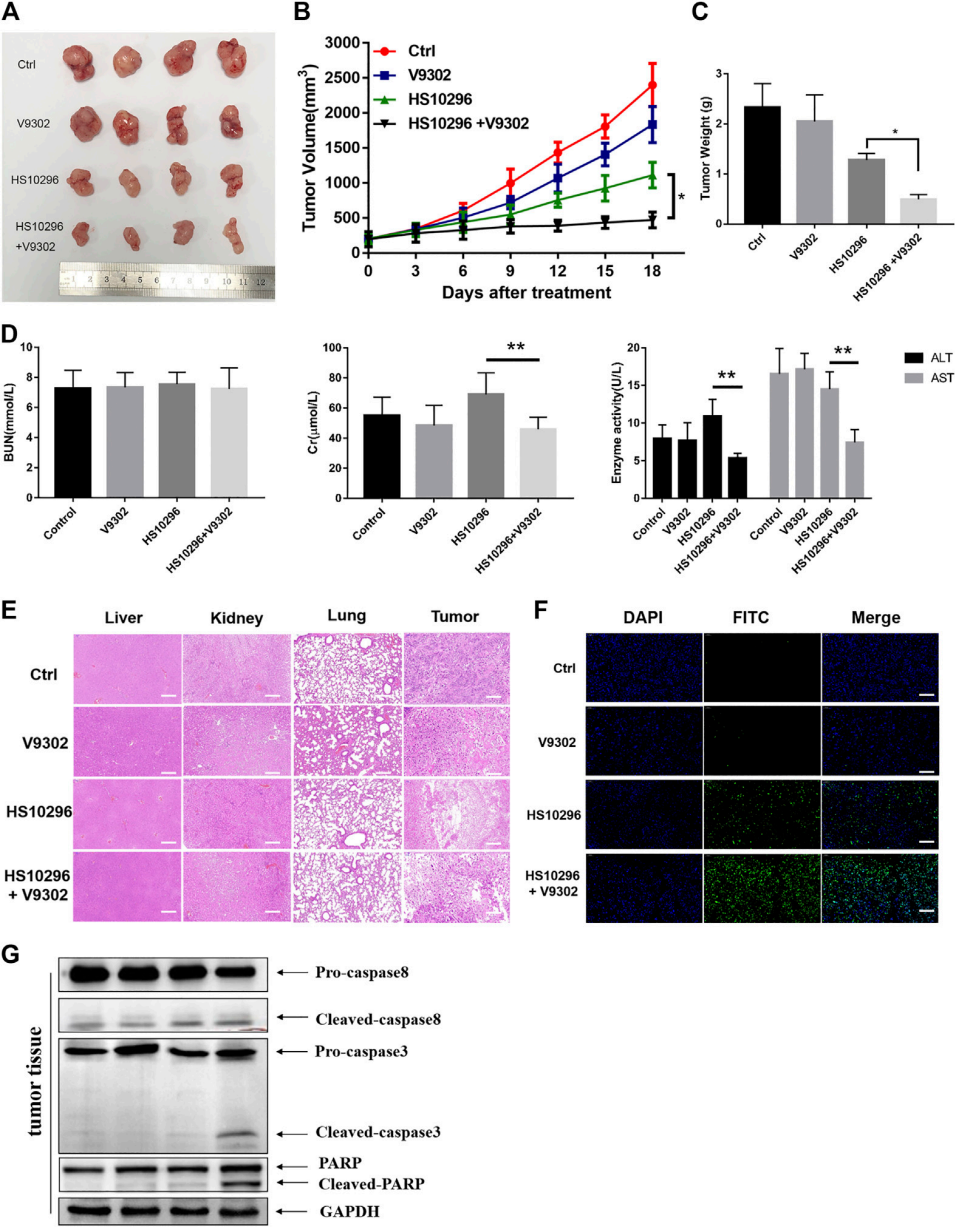
V9302 enhanced the antitumor effect of Almonertinib *in vivo*. **(A)** Representative tumors from each treatment group. **(B)** Tumor weight of the mice. **(C)** Tumor volume of the mice. **(D)** Evaluation of toxicity *in vivo* in nude mice. AST, ALT, BUN and Cr of serum were measured by assay kits, respectively. **(E)** H&E-stained sections of the tumor, liver, lung and kidney from the mice after treatment. **(F)** TUNEL was used to detect apoptosis in tumor tissues. **(G)** Protein expression of caspase8, caspase3 and PARP were detected by WB in tumor tissues. Significance: *p < 0.05, **p < 0.01, ***p < 0.001.

To determine whether V9302 enhanced the efficacy of Almonertinib on NSCLC cell *in vivo*, a tumor-bearing nude mouse model was established using H1975 cells. Almonertinib and V9302 alone or a combination of the two drugs was treated to the tumor-bearing nude mice. V9302 alone had little effect on tumor growth, and Almonertinib alone could inhibit tumor growth to a greater extent than the blank group or the V9302 group. Compared with single-drug therapy, combined use of two drugs significantly inhibited tumor growth (Figures 5A,B). Additionally, the combined treatment of Almonertinib and V9302 could significantly decrease tumor weight (Figure 5C). We subsequently evaluated the toxicity of Almonertinib and V9302 alone or in combination on the liver, lung and kidney of nude mice. Two indexes of serum ALT and AST were used to evaluate liver function, and two indexes of BUN and Cr were used to evaluate renal function in this study. Serum test results showed that neither single or combined use of Almonertinib and V9302 caused liver nor kidney toxicity (Figure 5D). Meanwhile, Hematoxylin and eosin stain (HE) staining showed that neither single or combined use of Almonertinib and V9302 caused damage to liver, lung or kidney tissue. While, Almonertinib and V9302 combined group and Almonertinib group could cause tumors tissue damage compared with the blank control group (Figure 5E). Finally, TUNEL staining was used to detect the nuclear DNA fragmentation in tissue and cells in the early stages of apoptosis. Since normal or proliferating cells have almost no DNA fragmentation, causing them rarely be stained. As a result, the green fluorescence of the Almonertinib and V9302 combination group was significantly enhanced than Almonertinib single drug group, indicating that V9302 could enhance the effect of Almonertinib on inducing tumor cell apoptosis (Figure 5F). Apoptosis markers, including the cleavage of caspase 8 and caspase 3 and the enzymolysis of the substrate PARP, were detected in tumor tissue, it is showed that the WB results were consistent with the experimental result *in vitro* (Figure 5G). The above results indicated that V9302 could enhance the anti-tumor effect of Almonertinib *in vivo*, and the two drugs had no obvious liver and renal toxicity whether used alone or in combination.

### Effect of V9302 on Almonertinib-Induced Autophage in NSCLC Cells

**FIGURE 6:**
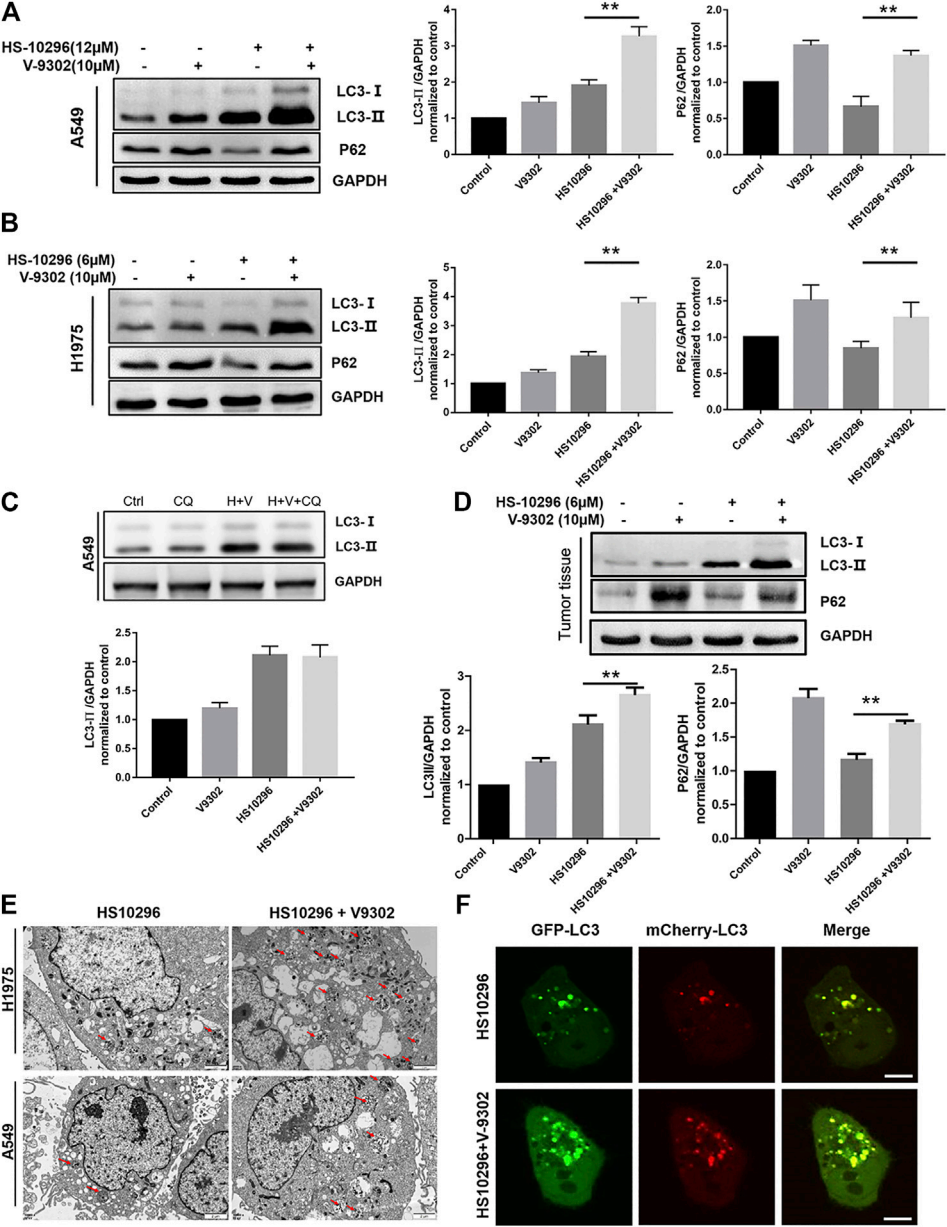
Effect of V9302 on Almonertinib-induced autophagy. **(A,B)** Effect of different drug groups on protein expression of autophagy marker protein LC3-II and P62 in cell lines by WB. **(C)** Almonertinib alone or combined with chloroquine to observe the effect on the protein level of LC3-II. **(D)**. Effect of different drug groups on protein expression of autophagy marker protein LC3-II and P62 in tumor tissues. **(E)** A549 (H1975) cells were treated with 12 μM (6 μM) Almonertinib alone or in combination with 10 μM V9302 for 12 h, then fixed and observed with an electron microscope. **(F)** mCherry-GFP-LC3B was used to detect the autophagy flux level of Almonertinib combined with V9302 in H1975 cells for 12 h. Significance: *p < 0.05, **p < 0.01.

α-KG, an intermediate in the Krebs cycle produced by glutamine metabolism, was reported to activate the mTORC1 pathway and inhibit macroautophagy (Duran et al., 2012). Autophagy is a catabolic process regulated by the mTORC1 pathway. The lysosomal degradation of cell components provides cells with recirculating nutrients through this process (Shacka et al., 2006; Jiang et al., 2015; Dizon et al., 2016). EGFR-TKIs have also been validated to activate autophagy in NSCLC and other cancer cells (Shacka et al., 2006). We further investigated the effects of glutamine transporter inhibition on HS1029 levels in this study. WB was first used to detect the protein expression of autophagy-related proteins LC3 and p62 in different administration groups. There are two main forms of LC3 protein, namely LC3-I and LC3-II, which is one of the hallmarks to detect autophagy. When autophagy occurs, cytoplasma-type LC3 (LC3-I) would be transformed into membrane-type LC3 (LC3-II), participating in the biosynthesis of autophagosomes (Nakatogawa et al., 2009). As shown in Figures 6A,B, the protein expression of LC3-II in the V9302 and Almonertinib combination group and Almonertinib group was significantly higher in A549 and H1975 cells, indicating that a combination of V9302 and Almonertinib can induce the formation of autophagosome. However, whether there was any effect on the degradation of autophagosomes, it was necessary to further combine with chloroquine (lysosomal inhibitor) to observe the changes of LC3-II after combining with chloroquine. As shown in Figure 6C the LC3-II level of the Almonertinib + V9302 + CQ group was not further increased compared with the Almonertinib + V9302 group, indicating that the Almonertinib + V9302 group could inhibit the degradation of autophagosomes and prevent the binding between autophagosomes and lysosomes. p62 is the substrate protein of autophagy, which can be degraded during autophagy. As shown in Figures 6A,B, the protein expression of p62 in the Almonertinib + V9302 group was higher than that in the Almonertinib group, indicating that the autophagic flux was blocked and further validating that Almonertinib + V9302 group could increase the synthesis of autophagosomes and inhibit the degradation of autophagosomes. The detection results of related autophagy indicators by WB in tumor tissue were also consistent with the cell experimental results *in vitro* (Figure 6D).

TEM was further used to observe the changes of autophagosomes in cells after different drug treatment. As shown in Figure 6E the number of autophagosomes in H1975 cells in the Almonertinib + V9302 group was significantly increased compared to the Almonertinib group. Similarly, we also observed this phenomenon in A549 cells, further validating Almonertinib + V9302 could increase the synthesis of autophagosomes. Laser confocal microscope was used to observe the changes in intracellular autophagic flux after treatment with different drug groups. GFP-mCherry-LC3B tandem fluorescent protein is a fusion protein specifically used to detect the level of autophagic flux (Singh et al., 2014). In the activation of autophagic flux, cellular red and green fluorescence would appear dot-like aggregation, that is, the green and red fluorescence increased. Meanwhile, the number of red fluorescent bright spot (autophagolysosome) and yellow fluorescent bright spot (autophagosome) would increase in the image synthesized by the red fluorescence and green fluorescence. As shown in Figure 6F, the red and green fluorescent bright spots in H1975 cells transiently transfected with mCherry-GFP-LC3B was increased compared with the Almonertinib group and Almonertinib + V9302 group. In addition, the number of yellow fluorescent bright spots (autophagosomes) was significantly increased in the synthesized images of red and green fluorescence, without red fluorescent bright spots (autophagolysosomes), indicating that the synthesis of autophagosome was increased and autophagosomes did not bind to lysosomes to form autophagolysosomes. The above three sets of results suggested that V9302 enhanced the synthesis of Almonertinib-induced autophagosomes, and simultaneously prevented the degradation of autophagosomes.

### Effects of Chloroquine Treatment on the Proliferation and Apoptosis of A549 Cells Co-treated With V9302 and Almonertinib

**FIGURE 7:**
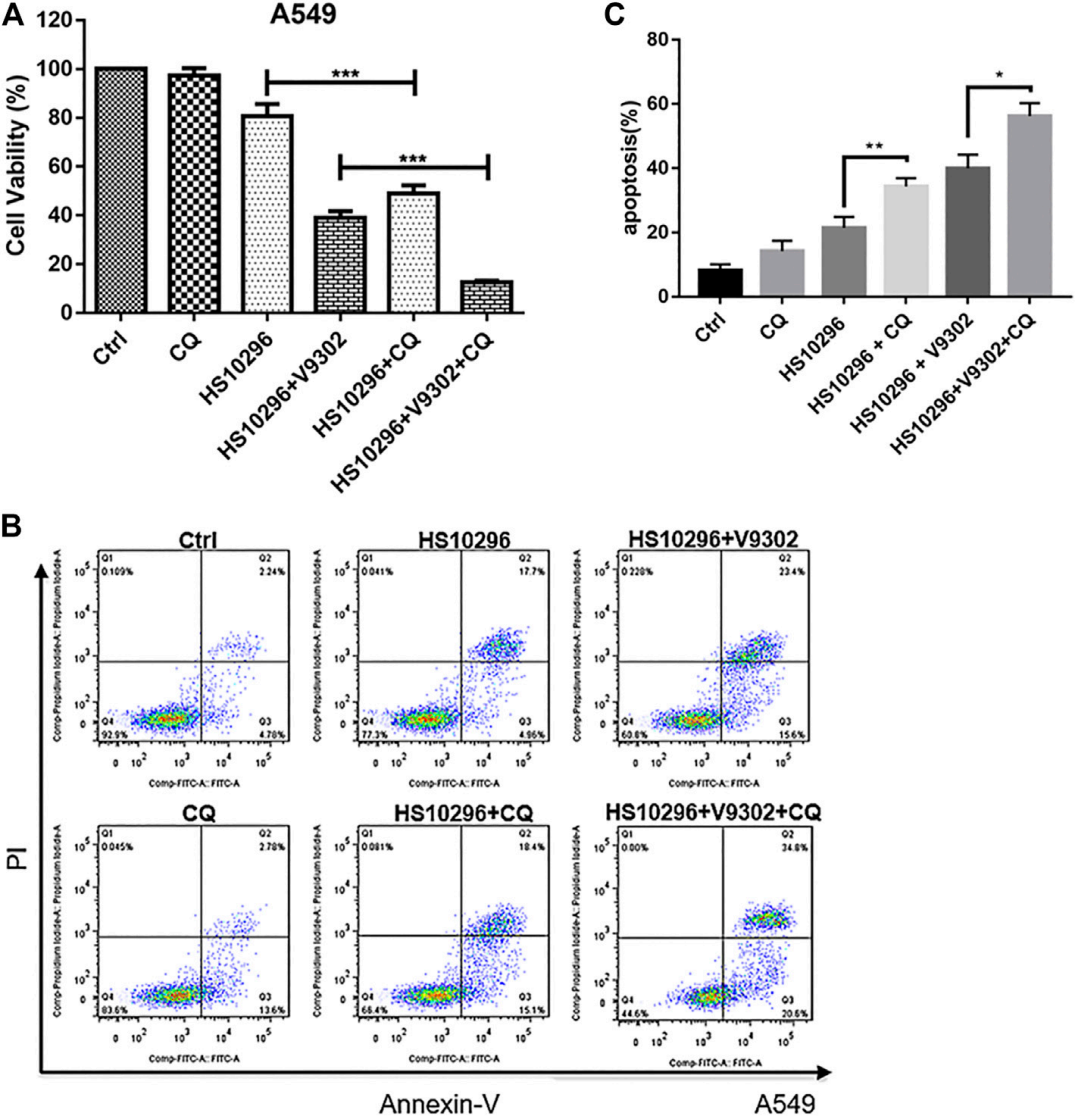
CQ increased the proliferation inhibitory effect of Almonertinib combined with V9302 on A549 cells and induces apoptosis. **(A)** CCK8 assay was used to determine the effect of CQ on the proliferation inhibition of Almonertinib and V9302 co-treated A549 cells. **(B,C)** Double staining assay was used to detect the effect of CQ on the induction of apoptosis of A549 cells treated with Almonertinib and V9302. Significance: *p < 0.05, **p < 0.01,***p < 0.001.

To assess the relationship between autophagy inhibition and apoptosis induction in NSCLC caused by the combination of V9302 and Almonertinib, 15 μM CQ combined with Almonertinib (8 μM) or Almonertinib (8 μM) + V9302 (10 μM) were used to treat A549 cells to investigate the effect on cell proliferation and apoptosis. CCK8 assay suggested that the cell viability rate of the Almonertinib + CQ group was significantly lower than that of the Almonertinib group (*p* < 0.001), and the cell viability rate of the Almonertinib + V9302 + CQ group was also significantly lower than that of the Almonertinib + V9302 group (*p* < 0.001). Meanwhile, the apoptosis rate of Almonertinib group or Almonertinib + V9302 group after CQ treatment was significantly higher than that of Almonertinib group or Almonertinib + V9302 group without CQ treatment. As previous results, we know Almonertinib could induce apoptosis. However, surprisingly, the addition of autophagy inhibitor CQ induced apoptosis instead of rescuing cells from apoptosis (Figures 7A–C), suggesting that Almonertinib-induced autophagy may play a role in “protecting” cells. When Almonertinib combined with V9302 to inhibit autophagy, the “protection” of autophagy may be broken, thereby inducing more apoptosis.

## Discussion

It has been reported that there were approximately 18.1 million new cancer cases and 9.6 million cancer deaths globally in 2018. The number of deaths related to lung cancer is approximately 1.8 million, accounting for nearly one-fifth of all cancer-related deaths (Yun et al., 2009), severely affecting human health. NSCLC is the most important pathological subtype of lung cancer (Rotow and Bivona, 2017). In North America and Europe, only 10–20% of NSCLC patients have activated EGFR mutations (Westover et al., 2018), which rises to 60% in Asian patients (Nahar et al., 2018). Compared with standard chemotherapy, the administration of TKI therapy that specifically targets EGFR to treat EGFR-mutant lung cancer can prolong PFS and improve the quality of life (Schrank et al., 2018). However, patient’s response to these drugs is generally transient, and most patients develop secondary resistance after a year. At present, drug development for secondary resistance is mainly based on resistance mechanisms such as gene mutation, amplification, signal activation and histological changes. Due to the heterogeneity of drug-resistant cells and the diversity of molecular phenotypes, the efficacy of these reversal drugs fail to meet people’s expectations. After reviewing the literature, we found that the abnormal metabolism of tumor was closely associated with the drug resistance of tumor cells (Ali et al., 2018). And our team has been engaged in the study of targeted energy metabolism to reverse drug resistance or to increase the sensitivity of chemotherapeutics. First, we investigated the changes of energy metabolism-related transport channels after Almonertinib intervention at half-inhibitory concentration in H1975 cells through proteomics, revealing that SLC1A5 was induced to up-regulate. This result suggested that SLC1A5 may play an important role in the sensitivity of NSCLC to Almonertinib.

Lung cancer cells are addicted to glutamine to meet their energy requirements for rapid proliferation (Wang et al., 2015). To validate the dependence of NSCLC cells on glutamine, we first examined the proliferative inhibition of two cell lines under glutamine deficiency. As a result, glutamine deficiency inhibited the proliferation of lung cancer cells. Similarly, both pharmacological and genetic inhibition of SLC1A5 suppressed the proliferation of NSCLC non-small cell lung cancer, which proved the importance of glutamine for NSCLC.

Many studies have suggested the role of metabolic molecules in tumor growth and reversing tumor resistance. In this study, we used the third-generation EGFR-TKI Almonertinib in combination with SLC1A5 inhibitor to investigate the changes in the sensitivity of NSCLC cells to Almonertinib. As a result, the CI of the combined drug group was less than one, suggesting that the SLC1A5 inhibitor V9302 played a synergistic role with Almonertinib to inhibit the proliferation of H1975 and A549 cells. According to our findings, the IC50 of Almonertinib in A549 cells was 18.47 µM. The IC50 was 6.19 µM after the combination of V9302, which was decreased by 66.49%. Similarly, in H1975 cells, the IC50 of Almonertinib was 8.33 µM. And the IC50 was 4.89 µM after combination with V9302, which was decreased by 41.29%. Therefore, we conclude that SLC1A5 inhibition could significantly decrease the dosage of Almonertinib.

To further confirm whether the cell death was due to apoptosis, we tested the cleavage of several apoptosis-related proteins and found that Caspase 3 and Caspase 8 were both activated. Caspase is a “key regulator” in the apoptosis signaling pathway. Apoptosis is mainly induced through the mitochondrial pathway and the death receptor pathway, both of which are Caspase-dependent. The morphological manifestations of apoptosis are chromatin condensation, nuclear division, cell membrane invagination to form apoptotic bodies and cell shrinkage. Meanwhile, the experimental results also showed that PARP also underwent enzymatic hydrolysis. The proteolysis of PARP is a molecular event activated by cysteine protease and is an early molecular marker of programmed cell death. TEM was used to observe the submicroscopic structure of cells to determine the form of death. By observing the intracellular structure, we found that the combination group had obvious apoptosis characteristics. The above results together indicate that V9302 can increase the Almonertinib-induced apoptosis of NSCLC cells.

Autophagy plays a “double-edged sword” in tumor progression. Autophagy can not only eliminate misfolded proteins or damaged organelles to maintain tumor growth, but induce autophagic programmed cell death in tumor cells (Ashrafizadeh et al., 2020; Maiti and Hait, 2021). Autophagy is a dynamic process, including the formation of phagosomes and autophagosomes, the combination of autophagosomes and lysosomes to form autophagolysosomes and the degradation of autophagolysosomes. CQ is an autophagolysosome inhibitor, which can block the degradation of autophagolysosomes. At this time, the detected changes in LC3B-II represent the level of autophagosomes. If the autophagic flux is activated, the level of LC3B-II would continue to increase after using CQ; if the autophagic flux is blocked, the level of LC3B-II would not increase after using CQ. In this study, the level of LC3B-II was changed after using CQ in the Almonertinib and V9302 combination group, indicating that the autophagic flux was blocked in the Almonertinib and V9302 combination group. We subsequently discussed the correlation between autophagy and apoptosis. Surprisingly, the combination of CQ in both Almonertinib single group or the V9302 combination group led to significantly suppressed cell proliferation rate and induced more apoptosis. Since Almonertinib could induce autophagy, the combination of autophagy inhibitors significantly induced more apoptosis instead of protecting cells, suggesting that Almonertinib-induced autophagy may inhibit cell apoptosis, and the combination of V9302 and Almonertinib may be induces cell death by inhibiting autophagy. Unfortunately, in this study, we did not further investigate the effect on apoptosis by interfering with autophagy-related genes, which is also the direction of subsequent study.

Compared with targeted signal transduction pathways, strategies targeting key metabolic molecules are unlikely to lead to drug resistance, because it is relatively easier for cancer cells to compensate within the signal transduction system, while a lack of necessary metabolic pathways is not easy to compensate (Silva et al., 2016). In this study, we found that SLC1A5 was a key glutamine transporter, and its protein expression was up-regulated after Almonertinib stimulation in H1975 cells. We also found that inhibition of SLC1A5 enhanced the inhibitory effect of Almonertinib on NSCLC cells, which supported the idea of targeting tumor metabolic pathways to improve drug sensitivity. Almonertinib exerted a poor effect on EGFR WT NSCLC but had the best effect on EGFR mutant NSCLC. In this study, SLC1A5 inhibition not only enhanced the anti-tumor effect of Almonertinib on EGFR mutant cell lines (H1975), but greatly improved the anti-tumor effect of Almonertinib on EGFR WT cell lines (A549). Therefore, the present finding suggested that SLC1A5 may be an important candidate target to improve the application of Almonertinib in NSCLC regardless of EGFR status.

In summary, inhibition of glutamine transporter can enhance the effect of Almonertinib-induced apoptosis in NSCLC cells. We also found that Almonertinib enhanced the anti-tumor effect of Almonertinib *in vivo*. Noteworthy, Almonertinib showed no obvious toxicity on liver, kidney or lung of nude mice. This provides a theoretical basis to improve the sensitivity of NSCLC patients to HS1029 after marketing. Unfortunately, we failed to further explore the signaling pathway in this study, therefore, we will explore the related mechanisms in future research.

## Conclusion

Inhibition of SLC1A5 can enhance the anti-tumor effect of Almonertinib in NSCLC both *in vitro* and *in vivo* (*Graphical Abstract Image*). SLC1A5 may become an attractive target to improve the sensitivity of NSCLC to EGFR-TKIs.

## Data Availability

The raw data supporting the conclusion of this article will be made available by the authors, without undue reservation, to any qualified researcher.
