# Supplementary figures and images for: Restricting Glutamine Uptake Enhances NSCLC Sensitivity to Third-Generation EGFR-TKI Almonertinib
Source: Front Pharmacol. 2021 May 14;12:671328. doi: 10.3389/fphar.2021.671328 (PMC8161200; doi:10.3389/fphar.2021.671328)

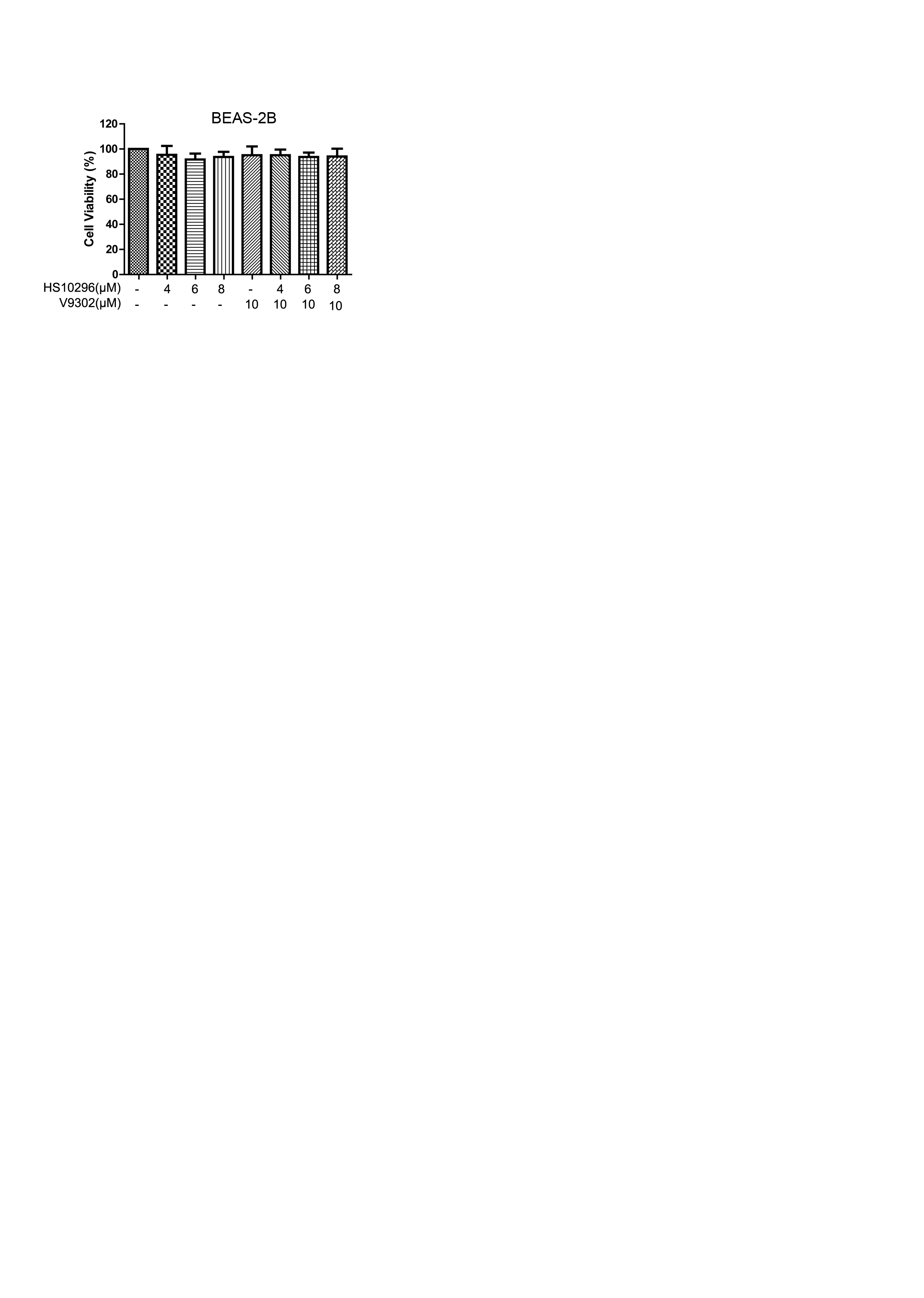

Supplement: Supplementary file 1 [file Image1.TIF]
